# Economic Burden of Tuberculosis in Iran: A Nationwide Analysis by Drug Resistance and Cost Components

**DOI:** 10.34172/jrhs.11215

**Published:** 2025-09-15

**Authors:** Masoud Arefnezhad, Mahshid Nasehi, Aliakbar Fazaeli, Saeed Sharafi, Rajabali Daroudi

**Affiliations:** ^1^Department of Health Management, Policy and Economics, School of Public Health, Tehran University of Medical Sciences, Tehran, Iran; ^2^Department of Epidemiology, School of Public Health, Iran University of Medical Sciences, Tehran, Iran; ^3^National TB and Leprosy Control Department, Ministry of Health and Medical Education, Tehran, Iran

**Keywords:** Tuberculosis, Multidrug-resistant, Extensively drug-resistant, Cost of illness, Iran

## Abstract

**Background:**

Tuberculosis (TB) remains a significant global health crisis, regaining its status as the leading cause of death in 2023. Quantifying its economic burden is essential for crafting effective public health strategies. This study aimed to estimate the economic burden of TB in Iran.

**Study Design:**

This study employed a cross-sectional design.

**Methods:**

A prevalence-based approach was used to estimate the economic burden of TB in Iran, accounting for cost variations across TB types and cost categories. Costs were categorized as direct medical, direct non-medical, and indirect, and were calculated for suspected TB patients as well as for those with drug-sensitive TB, multidrug-resistant TB, and extensively drug-resistant TB. Data were extracted from various sources, including the National Tuberculosis Registration System, national TB diagnosis and treatment guidelines in Iran, official medical service tariffs, and previous studies.

**Results:**

Of 210,544 individuals screened, 7,221 were diagnosed with TB, of whom 81.0% had pulmonary TB and 19.0% had extrapulmonary TB. Drug-sensitive TB accounted for 99.4% of cases, multidrug-resistant TB 0.6%, and extensively drug-resistant TB 0.0%. Diagnostic costs represented 48.0% of the total economic burden (approximately Int’l$4.71 million), while post-diagnosis costs totaled Int’l$5.15 million. Overall, economic burden, including all diagnostic and treatment expenses, amounted to approximately Int’l$9.86 million.

**Conclusion:**

This study underscores the significant economic burden of TB in Iran, encompassing both pre-diagnosis and post-diagnosis expenses, with direct medical costs representing the largest component. Effective healthcare strategies and comprehensive public health approaches are crucial to reducing these costs and improving patient outcomes.

## Background

 Tuberculosis (TB), a preventable and largely curable disease, re-emerged in 2023 as the leading cause of death from a single infectious agent worldwide^1^. Global TB incidence has increased steadily, with an estimated 10.8 million people developing TB in 2023, compared to 10.6 million in 2022 and 10.3 million in 2021, reversing prior gains. Although the incidence rate rose by only 0.2% between 2022 and 2023, this followed a sharper 2.2% increase in the previous year.^1^

 Beyond its health impacts, TB imposes a considerable financial and social burden on patients, families, and communities. It disproportionately affects individuals aged 15–54, a period of peak economic productivity.^2^ Illness often leads to reduced work capacity and generates indirect costs such as caregiver time and lost income. Direct treatment costs also place pressure on patients, governments, and insurance systems.^3^ Over the past two decades, several studies have highlighted TB’s role in undermining economic stability by reducing labor force participation, increasing health expenditures, and disrupting household finances.^4^ Catastrophic health spending is common among TB patients, sometimes leading to impoverishment.^5^ Additionally, TB-related stigma can increase social isolation and economic vulnerability.^6^

 Iran, located in the Eastern Mediterranean Region and bordering high-burden countries such as Afghanistan and Pakistan, launched its National TB Control Program in 1996. As a result, incidence has declined from 34 per 100,000 in the 1990s to 11 per 100,000 in 2023.^1^ However, the economic burden of TB remains considerable due to persistent treatment costs and productivity losses.^7^ Although TB diagnosis and treatment are provided free of charge in Iran, indirect costs such as transportation, lost earnings, and out-of-pocket medical expenses continue to impose a significant strain on many households.^8^

 Comprehensive assessment of TB’s economic burden is essential for health system planning, monitoring progress toward national and global targets, and informing financial protection strategies.^9^ While international evidence highlights TB’s economic impact,^6^ recent nationally representative estimates from Iran, particularly those disaggregating costs by drug resistance status, are lacking.

 Previous Iranian studies^10^ have largely focused on clinical outcomes or subnational cost analyses, offering limited insight into national-level financial impact. Moreover, few have examined the burden in terms of both direct (medical and non-medical) and indirect costs, or used standardized measures such as purchasing power parity (PPP) to facilitate cross-country comparison.

 This study aimed to address these gaps by providing the first nationally representative estimate of TB’s economic burden in Iran for 2024, disaggregated by drug resistance type, drug-sensitive TB (DS-TB), multidrug-resistant TB (MDR-TB), and extensively drug-resistant TB (XDR-TB). Using a prevalence-based approach and a societal perspective, the study incorporates real-world data, includes both pre- and post-diagnosis costs, and applies inflation-adjusted PPP values. The findings offer robust, policy-relevant insights to guide TB-related resource allocation, enhance financial protection, and enable regional and international comparisons across low- and middle-income countries with similar contexts.

## Methods

###  Study design

 This study employed a prevalence-based approach to estimate the economic burden of TB in Iran for the year 2024. The average cost per patient was calculated by considering the cost category, then multiplying it by the total number of patients to estimate the overall economic burden.

###  Prevalence data 

 The National Tuberculosis Registration System of Iran served as the primary source for disease prevalence data, a comprehensive database that plays a crucial role in the country’s TB control and prevention efforts. The registry collects data from public and private healthcare facilities, laboratories, and healthcare providers. Suspected TB cases are first reported to TB laboratories in counties, which then forward the data to the National TB and Leprosy Control Department at the Ministry of Health. This centralized system is designed to provide a relatively comprehensive overview of TB prevalence and distribution nationwide.

 The registry contains detailed information on TB patients, including their demographics, diagnostic results, treatment history, and disease progression. All data are meticulously recorded and reviewed by district coordinators before being entered into the registry, ensuring accuracy and consistency of the records. Within the registry, TB cases are categorized into different groups based on disease type and drug resistance patterns. These groups typically include DS-TB, MDR-TB, and XDR-TB. This classification aids in tailoring treatment protocols and facilitates optimal resource allocation.

###  Data validation and quality assurance

 To ensure the accuracy and reliability of the data, multiple validation steps were undertaken. The primary data source for this study was the Iranian National Tuberculosis Registry, which is managed by the Ministry of Health and Medical Education. This nationwide, mandatory reporting system collects patient information from public and private healthcare facilities, laboratories, and clinics across the country.

 Before conducting the analysis, the dataset underwent careful cleaning. Records with missing or inconsistent information, such as unknown diagnosis type, implausible age, or incomplete treatment data, were excluded. Only complete and valid records from the year 2024 were retained for the final analysis. The registry applies regular quality control procedures, including automatic checks for duplicate entries, consistency verification by district-level TB officers, and periodic audits conducted by provincial supervisors. Moreover, data entry is cross-checked and confirmed at the district level before final submission to the national system.

 Although all efforts were made to maintain data quality, the possibility of underreporting cannot be entirely ruled out. However, a published evaluation of Iran’s communicable disease surveillance system estimated an underreporting rate of approximately 13%, indicating that about 87% of actual TB cases are captured. This suggests an acceptable level of completeness for national burden estimation.^1^

###  Expert opinion and parameter estimation

 To supplement empirical data, several parameters, including the frequency of healthcare service utilization, were estimated through consultation with an expert panel. This panel comprised seven professionals with relevant expertise in TB management, health economics, and public health policy, including infectious disease specialists and senior officials from the Communicable Diseases Department of the Ministry of Health in Iran. Although a formal Delphi technique was not employed, structured discussions were conducted to reach consensus estimates that reflect current clinical practices and operational realities within the Iranian healthcare system. This pragmatic approach ensured that expert-derived parameters were robust, contextually relevant and enhanced the validity of our economic burden analysis.

###  Cost estimation 

 Costs associated with TB were estimated from a societal perspective, encompassing both pre-diagnosis and post-diagnosis expenses. These costs were classified into diagnostic expenses for individuals with suspected TB and treatment expenditures for confirmed cases across different resistance patterns, including DS-TB, MDR-TB, and XDR-TB. Further, costs were categorized into direct medical costs, direct non-medical costs, and indirect costs.

 Cost calculations were primarily based on private sector medical tariffs, which are standardized, publicly available, and reflect the actual economic value of services rendered. Although most TB patients in Iran are treated within the public healthcare system, where services are subsidized or provided free of charge, public sector tariffs lack transparency and are not consistently updated. Using private sector tariffs allows better capture of out-of-pocket expenditures and supplementary services frequently utilized by patients. To mitigate potential overestimation, sensitivity analyses using alternative cost scenarios based on estimated public sector tariffs were conducted. These analyses confirmed the validity of the overall economic burden estimates.

###  Direct medical costs

 Direct medical costs include diagnostic procedures for suspected TB cases such as chest X-Ray (CXR), sputum microscopy (SM), Gene Xpert (GX), culture tests, and real-time polymerase chain reaction (RT-PCR), as well as outpatient and inpatient treatment costs for confirmed TB patients, including doctor visits, medications, directly observed treatment short-course (DOTS), and hospitalization.

 A bottom-up costing approach was applied to estimate direct medical costs by different types of TB, using the following equation:


$$Direct\;Medical\;Cost=\sum_{i=1}^{n}\sum_{g=1}^{4}N_{ig}*F_{ig}*C_{i}$$

where *i *denotes the type of medical service, *g* refers to the various types of TB (i.e., pre-diagnosis, DS-TB, MDR-TB, and XDR-TB). *N* represents the number of patients utilizing each service for the respective TB type, *F* indicates the frequency of utilization of each service across these types, and *C* signifies the unit cost of each service.

 The type of services utilized by patients, the number of patients using each service, and the average frequency of receiving each service were derived from current diagnosis and treatment guidelines in Iran^2^, data from the TB registration system, and expert opinions. Unit costs for diagnostic services, physician visits, and hospital stays were determined using approved private-sector medical service tariffs set by the Iranian Ministry of Health. International reference prices were used for estimating medication costs.

 The cost of implementing the DOTS strategy in this study reflects payments made to healthcare workers for directly observing patients during the administration of anti-TB medications. Two complementary methods were used to estimate this cost. First, we applied national tariffs set by the Ministry of Health and Medical Education, multiplying the unit cost of one DOTS visit by the average number of DOTS encounters per patient, as outlined in national treatment protocols. Second, to enhance accuracy, a time-driven micro-costing approach was used. Based on expert consensus, the average duration of each DOTS visit was estimated at 15 minutes. This duration was then multiplied by the minute-based wage of community health workers (Behvarz), calculated from their hourly salary data adjusted to 2024 values. Only cost components directly associated with DOTS supervision were included, while unrelated services (e.g., diagnostics or consultations) were excluded. This hybrid costing approach strengthens the validity of the estimates and is consistent with previous cost-of-illness studies in Iran.^6^

###  Direct non-medical costs

 Direct non-medical costs encompass transportation, accommodation, food, and medical equipment expenses incurred by patients. Transportation costs account for patient travel to and from healthcare facilities, while accommodation costs cover stays near treatment centers, especially for individuals from remote areas. Food and equipment costs account for additional dietary needs and necessary medical devices during treatment. These cost estimates were primarily drawn from a previously published Iranian study,^10^ with values adjusted to 2024 prices using official inflation rates. To ensure relevance and accuracy, the adjusted costs were cross-validated against recent national health reports and expert consultations within the Iranian TB control program. Moreover, deterministic sensitivity analyses were conducted to assess the impact of potential variations in non-medical costs on the overall economic burden.

 To calculate the total direct non-medical costs across different resistance patterns, the average cost per patient was multiplied by the total number of patients for each type of TB.


$$Direct\;Non-medical\;Cost=\sum_{i=1}^{4}\sum_{g=1}^{4}N_{ig}*C_{i}$$

where *i* represents the category of non-medical costs (i.e., transportation, accommodation, food, and equipment), *g* denotes the TP type (i.e., pre-diagnosis, DS-TB, MDR-TB, and XDR-TB), *N* signifies the number of patients within each TB category, and *C* represents the average cost per patient.

###  Indirect costs 

 Indirect costs associated with TB included productivity losses from both temporary absenteeism and premature mortality. For absenteeism, the number of workdays lost during diagnosis, treatment, and recovery was estimated: 60 days for DS-TB and 180 days for MDR/XDR-TB. These estimates were derived from a cost-of-illness study in Thailand^4^ and validated by a panel of Iranian TB experts to ensure contextual relevance.

 Beyond absenteeism, long-term productivity losses due to premature death were modeled using a demographic-specific approach. For each deceased patient, we applied age- and sex-specific employment rates derived from national labor force data, alongside the 2024 minimum daily wage published by the Statistical Center of Iran.^5^ Lost future income was estimated over the expected remaining lifetime and discounted at an annual rate of 5%, consistent with international economic evaluation standards. This dual approach provided a more precise and realistic estimation of the indirect economic burden of TB in Iran.


$$Absenteeism\;Cost=\left(\sum_{i=1}^{n}\sum_{g=1}^{2}M_{ig}*P_{ig}*W_{i}*N_{ig}\right)$$

where *i *represents the age group, *g* the gender, *M* the average workdays missed per TB patient, *P* the workforce participation rate, *W* the daily wage, and *N* the number of TB patients. In this model, productivity losses related to caregivers were excluded.

 Premature mortality costs represent the economic value of lost income due to premature deaths caused by a particular disease or health condition. The human capital approach was employed to estimate premature mortality costs using the following equation:


$$Premature\;Mortality\;Cost=(\sum_{i=1}^{n}\sum_{g=1}^{2}\sum_{le=1}^{60}AA_{ig}*N_{igc}*P_{ig})/{\left(1+DR\right)}^{t}$$

where *i* indicates age group, *g* gender, *AAI* average annual income or productivity value, *N* number of premature deaths, *P* workforce participation rate, *LE* remaining life expectancy, and *DR* discount rate. The number of TB-related deaths was obtained from the Iranian National Tuberculosis System, while remaining life expectancy was derived from national life tables.^5^ Future income was discounted to present value at a 5% annual discount rate. For international comparability, all costs were converted from Iranian Rials (IRR) to international dollars (I$) using the 2024 PPP conversion factor for Iran, as reported by the World Bank. The PPP conversion factor for 2024 was 117,170.2 IRR per international dollar.^11^

###  Uncertainty and sensitivity analysis 

 To address uncertainties stemming from input variability and model assumptions, we conducted both deterministic and probabilistic sensitivity analyses. A one-way sensitivity analysis was conducted on key parameters, including the average cost of hospitalization, the unit cost of diagnostic tests, daily productivity loss, and the discount rate applied to future productivity losses from premature mortality. In line with established recommendations from the World Health Organization (WHO)^7^ and the Second Panel on Cost-Effectiveness in Health and Medicine,^8^ discount rates of 3%, 5% (base case), and 7% were tested to evaluate their impact on indirect cost estimates.

 In addition, a probabilistic sensitivity analysis (PSA) was performed using Monte Carlo simulation (1,000 iterations). Appropriate probability distributions were assigned to input parameters: beta distributions for probabilities, gamma distributions for cost variables, and triangular distributions for durations. The PSA results are presented using 95% confidence intervals for the estimated total economic burden. These analyses enhanced the robustness of our findings by quantifying the potential variability in cost estimates under real-world uncertainty. All calculations and data analyses were performed using Microsoft Excel version 16.

## Results

###  Demographic and general information

**Figure 1:**
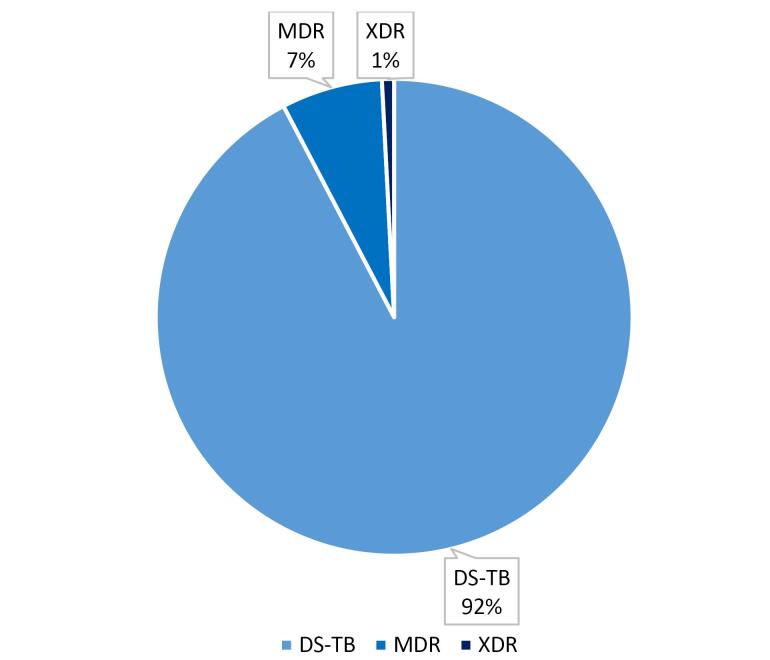
Distribution of Economic Burden by Type of Case, 2024 (N = 7,221)

 This study evaluated the economic burden of TB in Iran. In 2024, a total of 210,544 individuals with suspected TB symptoms sought medical care within the Iranian healthcare system. All were registered in the national TB database and underwent various diagnostic testing. Following the screening process, 7,221 individuals were diagnosed with TB. Among these, 81.0% were pulmonary TB, and 19.0% were extrapulmonary TB. DS-TB accounted for 99.4% of the diagnosed cases, while 0.6% were identified as MDRTB and 0.03% as XDR-TB as illustrated in Figure 1. In terms of gender, 57.0% of patients were male, and 43.0% were female. By nationality, 75.0% were Iranian and 25.0% non-Iranian. The majority of patients (70.0%) resided in urban areas, and 62.0% were married. The mean age of TB patients was 51.5 years overall, with Iranian patients averaging 54.1 years, while non-Iranian patients had a lower mean age of 43.6 years. The largest age group comprised individuals aged 65 years and older (29.0%). Table 1 presents detailed demographic and general characteristics of TB patients in Iran.

**Table 1:** Demographic and general information of TB patients in Iran, 2024

**Variables**	**Number**	**Percent**
Sex		
Male	4116	0.57
Female	3105	0.43
Nationality		
Iranian	5416	0.75
Non-Iranian	1805	0.25
Residence		
City	5062	0.70
Rural and countryside	2159	0.30
Marital status		
Married	4506	0.62
Single	1531	0.21
Divorced	217	0.03
Widowed	953	0.13
Unknown	14	0.01
Age group (year)		
< 15	188	0.03
15-24	758	0.11
25-34	1191	0.17
35-44	1069	0.15
45-54	895	0.12
55-64	1004	0.14
> 65	2116	0.29
Education level		
Illiterate	3033	0.42
Elementary school	1639	0.23
Middle school	1105	0.15
Secondary school/religious studies	996	0.14
Assistant to doctorate	440	0.06
Ph. D/Specialist and above	7	0.00
Type of disease		
Pulmonary TB	5819	0.81
Extra-pulmonary TB	1402	0.19
Drug resistance pattern		
Drug-sensitive TB	7174	0.99
Multi-drug-resistant TB	45	0.00
Extensively drug-resistant TB	2	0.00

*Note.* TB: Tuberculosis.

###  Diagnostic costs for suspected patients

**Figure 2:**
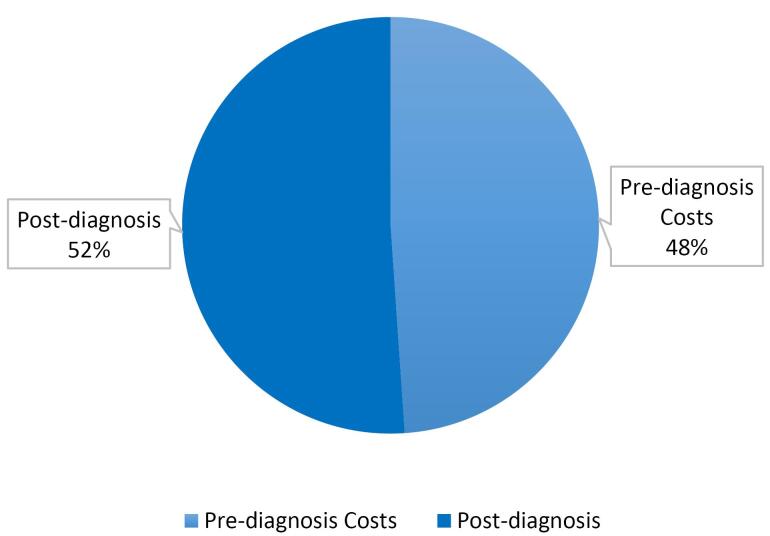
Distribution of economic burden by pre-diagnosis and post-diagnosis costs, 2024 (N = 210,544)

 As shown in Figure 2, diagnostic costs account for 48.0% of the total economic burden of TB in Iran, amounting to approximately Int’l$ 4.7 million (Table 2). The total pre-diagnosis cost for 210,544 individuals screened for TB in 2024 was estimated at PPP$4.7 million. These costs encompass direct medical, direct non-medical, and indirect components associated with diagnostic procedures prior to confirmed diagnosis.

**Table 2:** Pre-diagnosis costs for suspected TB patients (N = 210,544), Iran, 2024

**Cost type**				**Number of consumers**	**Services per Patient**	**Total services**	**Unit cost (PPP$)**	**Total cost (PPP$)**	**Percentage (%)**
Direct Costs	Medical costs	Diagnostic tests	Chest X-ray	4,595	1	4,595	2.05	9,419.75	0.00
			Sputum microscopy	210,544	2	421,088	2.17	913,760.96	0.19
			Gene Xpert	70,000	1	70,000	5.51	385,700.00	0.08
			Culture	31,741	1	31,741	5.85	185,684.85	0.04
			Regional focal point	8,422	1	8,422	1.60	13,474.82	0.00
			RT PCR	46,400	1	46,400	12.92	599,488.00	0.13
		Doctor’s Visits		210,544	1	210,544	1.06	223,176.64	0.05
	Non-medical costs	Transportation		210,544	1	210,544	0.27	56,846.88	0.01
Indirect costs	Productivity losses due to temporary disability			210,544	6	1,157,992	2.01	2,327,563.92	0.49
Total								4,715,115.82	1.00

*Note.* TB: Tuberculosis; PPP: Purchasing power parity; RT-PCR: Real-time polymerase chain reaction; (in International Dollars, PPP-adjusted).

 The largest share (49.0%) of this total cost was due to productivity losses from temporary disability, amounting to PPP$ 2,327,563.9. This reflects the significant economic impact of workdays lost while patients sought diagnosis and underwent preliminary evaluations.

 Among direct medical costs, sputum smear microscopy (SM) incurred the highest expenditure (PPP$913,760.9; 19.0%), followed by RT-PCR tests (PPP$599,488.0; 13.0%), and GX (PPP$385,700.0; 8.0%). Other diagnostic modalities, such as culture (PPP$185,684.9), regional focal point assessments (PPP$13,474.8), and CXRs (PPP$9,419.8), collectively accounted for a smaller proportion of total costs. Physician consultations, also classified as direct medical expenditures, totaled PPP$223,176.6 (5.0%), while transportation expenses— the main direct non-medical cost—were estimated at PPP$56,846.9 (1.0%) for all suspected patients.

 This cost breakdown highlights the substantial financial burden of the diagnostic phase of TB care, with indirect productivity losses and molecular diagnostics (RT-PCR and GX) as major contributors. The findings underscore the importance of optimizing diagnostic algorithms and expanding access to rapid, cost-effective testing methods to reduce unnecessary delays and associated economic losses.

###  Post-diagnosis costs incurred for tuberculosis patients

 As presented in Table 3, the post-diagnosis costs for 7,221 TB patients in Iran in 2024 totaled approximately PPP$5,152,603.5. These costs were categorized into direct medical, direct non-medical, and indirect components, stratified by TB resistance type: DS-TB, MDR-TB, and XDR-TB. Among the 7,174 DS-TB patients, the average post-diagnosis cost per patient was PPP$661.4, yielding a total cost of PPP$4,744,928.3. For MDR-TB (45 patients), the average cost rose significantly to PPP$ 8,085.1, resulting in a total of PPP$363,830.0. The highest per-patient costs were observed in XDR-TB (2 patients), with an average of PPP$21,922.1 and a combined total of PPP$43,846.2.

**Table 3:** Post-diagnosis Direct and Indirect Costs Incurred for TB Patients (n = 7,221), 2024 (in International Dollars, PPP-adjusted)

**Cost type**	**DS-TB (n=7174)**			**MDR-TB (n=45)**			**XDR-TB (n=2)**			**Overall**	
	**Average cost per patient (PPP$)**	**Total cost (PPP$)**	**Percentage** **(%)**	**Average cost per patient (PPP$)**	**Total cost (PPP$)**	**Percentage** **(%)**	**Average cost per patient (PPP$)**	**Total cost (PPP$)**	**Percentage** **(%)**	**Total cost (PPP$)**	**Percentage** **(%)**
**Direct costs**											
Medical costs											
Diagnostic tests	14.66	105,176.80	0.02	128.35	5,775.62	0.02	141.18	282.36	0.01	111,229.78	0.02
Doctor’s visits	8.51	61,065.09	0.01	28.73	1,292.76	0.00	31.60	63.20	0.00	62,421.05	0.01
Hospitalization	306.67	2,200,099.00	0.46	5,826.67	262,200.00	0.72	19,228.00	38,456.00	0.88	2,500,755.00	0.49
Medications	23.71	170,109.89	0.04	1,194.99	53,774.71	0.15	1,314.49	2,628.98	0.06	226,513.59	0.04
DOTS	47.50	340,765.00	0.07	190.00	8,550.00	0.02	209.00	418.00	0.01	349,733.00	0.07
Non-medical costs											
Transportation	15.03	107,832.19	0.02	33.37	1,501.61	0.00	61.17	122.35	0.00	109,457.15	0.02
Accommodation	16.13	115,708.65	0.02	35.81	1,611.28	0.00	65.65	131.29	0.00	117,451.21	0.02
Food and equipment	9.73	69,812.21	0.01	21.60	971.17	0.00	39.61	79.22	0.00	70,864.59	0.01
**Indirect costs**											
Productivity losses (temporary disability)	121.09	868,723.57	0.18	363.28	16,347.60	0.04	399.61	799.22	0.02	886,869.39	0.17
Productivity losses (premature heath)	98.36	705,637.93	0.15	262.30	11,803.24	0.03	432.78	865.57	0.02	718,305.74	0.14
Total	661.40	4,744,928.32	1.00	8,085.09	363,829.98	1.00	21,922.10	43,846.19	1.00	5,152,603.50	1.00

*Note*. PPP: Purchasing power parity; DS-TB: Drug-sensitive tuberculosis; MDR-TB: Multi-drug-resistant tuberculosis; XDR-TB: Extensively drug-resistant tuberculosis; DOTS: Directly observed treatment short-course.

 The largest contributor to direct medical costs was hospitalization, which alone accounted for PPP$2,500,755.0 (49.0% of the total), followed by DOTS (PPP$349,733.0) and medications (PPP$226,513.6). Diagnostic services, doctor consultations, and other clinical procedures accounted for smaller shares. Direct non-medical costs, including transportation (PPP$109,457.2), accommodation (PPP$117,451.2), and food and equipment (PPP$70,864.6), totaled approximately PPP$297,773.0, accounting for about 6.0% of the total post-diagnosis burden. Indirect costs were also substantial, primarily due to productivity losses during treatment and premature death. Temporary disability accounted for PPP$886,869.4 in losses, while premature mortality contributed an additional PPP$718,305.7, totaling PPP$1,605,175.1 (approximately 31.0% of the total post-diagnosis costs). This distribution highlights the disproportionately high economic burden imposed by drug-resistant TB cases and underscores the critical importance of early diagnosis, effective treatment, and targeted support for high-risk patients to reduce costs associated with advanced TB management.

###  Sensitivity analysis

 To assess the robustness of our cost estimates and address parameter uncertainty, both deterministic (one-way) and PSA were conducted. In the one-way sensitivity analysis, key cost drivers were varied within plausible ranges ( ± 20%) based on observed data variability and expert opinion. Parameters tested included the average hospitalization cost, daily productivity loss, and the prices of diagnostic tests such as sputum SM, GX, and CXR. The results revealed that the total economic burden of TB in Iran was most sensitive to variations in productivity losses from temporary disability and hospitalization costs. Specifically, a 20% increase in productivity loss led to an approximate 9.8% rise in total costs, while a similar increase in hospitalization expenses resulted in an 8.5% rise in overall expenditures. Conversely, diagnostic test costs showed comparatively minor effects, with less than 3.0% variation in total costs.

 A PSA using Monte Carlo simulation (1,000 iterations) was performed to capture simultaneous uncertainty across all major cost inputs. Appropriate probability distributions were applied: beta distributions for probabilities, gamma distributions for cost parameters, and triangular distributions for durations of absenteeism and treatment. The PSA yielded a mean total economic burden estimate of I$26.4 million, with a 95% confidence interval ranging from I$21.7 million to I$31.2 million. Overall, these sensitivity analyses underscore the pivotal role of indirect costs, particularly productivity losses, in shaping the economic burden of TB. This suggests that interventions reducing morbidity and absenteeism could yield substantial economic benefits. Moreover, the relatively narrow confidence intervals observed in PSA indicate that the model’s results are robust to plausible variations in key inputs, strengthening the validity of the study’s conclusions and their applicability to policy-making.

## Discussion

 This study assessed the economic burden of TB in Iran in 2024, with total expenditures estimated at approximately I$10.0 million, which included both pre-diagnosis and post-diagnosis costs. Pre-diagnosis costs, associated with screening 210,544 individuals, accounted for I$4.7 million (47.0%), while post-diagnosis costs for 7,221 confirmed TB cases amounted to I$ 5.3 million (53.0%). To ensure international comparability, all cost estimates were converted from IRR to $ using the 2024 PPP conversion factor of 117,170.2 IRR per I$, as reported by the World Bank.^11^ PPP-adjusted figures offer a more accurate reflection of resource utilization within the local economy, minimizing distortions from exchange rate fluctuations and enhancing the relevance of findings for global policy and economic evaluation.

 The average cost per patient for DS-TB in Iran was estimated at I$684.8 in 2024. With 7,174 DS-TB cases, the overall expenditure reached approximately I$4.9 million. Comparative analyses reveal striking differences in DS-TB treatment costs across income settings. High-income countries report^12^ a mean cost of US$14,659.0 per patient (based on a 2015 study), largely driven by advanced diagnostics, higher labor costs, and more comprehensive treatment services. In contrast, middle-income countries report an average cost of US$840.0 per case, while low- and lower-middle-income countries report an average of just US$273.0 per case. These differences reflect disparities in healthcare infrastructure, accessibility, and resource allocation. These comparisons underscore the importance of using PPP-based standardization to facilitate international policy evaluation and resource planning.^12^

 Notably, Indonesia stands out among middle-income countries with a higher average cost of US$ 2,150.0 per patient.^13^ European studies indicate a wide range of expenses, with Spain reporting TB treatment costs between €10,262.6 and €14,961.7, while Denmark documented €10,509.0.^14^ Conversely, lower costs in countries such as China (US$1,185.5)^15^ and the Democratic Republic of the Congo (US$400.0)^16^ highlight the impact of economic constraints. Furthermore, India’s 2024 review estimated an even lower DS-TB care cost of US$235.0 per patient.^17^ Similarly, in the Eastern Mediterranean region, low costs in Yemen at US$142.4^18^ and Pakistan at US$141.7 USD^19^ demonstrate significant economic limitations. African countries also show variation, with reported treatment costs of US$252.0 in Tanzania,^20^ US$256.6 in South Africa,^21^ and US$400.0 in the Democratic Republic of the Congo.^16^ These findings underscore the necessity for context-specific strategies, improved resource utilization, and stronger international collaboration to effectively reduce the economic burden of TB.

 The economic burden of MDR-TB in Iran is substantially higher than that of drug-susceptible TB. In 2024, the average cost per MDR-TB patient was estimated at I$8,108.5, leading to a total expenditure of I$364,283.6 for 45 diagnosed cases. This considerable per-patient cost reflects the extended treatment duration, higher medication prices, increased frequency of hospitalizations, and additional monitoring requirements associated with MDR-TB management. When adjusted using the PPP method, these figures provide a more accurate representation of the true economic impact on the Iranian health system and enable meaningful international comparisons.

 Comparative analysis of MDR-TB treatment costs across various countries reveals substantial disparities. In Tanzania, the cost per patient is significantly lower at $US650.0,^20^ while in the Democratic Republic of the Congo, it is US$1,224.0.^16^ South Africa reports a higher cost of US$6,771.9,^21^ reflecting its more advanced healthcare infrastructure. In Vietnam, the per-patient cost is US$4,302.0,^22^ while in Zimbabwe, it is US$3,569.0.^23^ China’s relatively high cost of US$8,266.0 per patient highlights the comprehensive nature of its healthcare services.^15^ Indonesia reports US$2,804.0 per patient,^13^ with Uganda^24^ and the Philippines^25^ reporting US$4,200.9 and US$2,357.0 per patient, respectively.

 European countries exhibit significantly higher treatment costs per patient, with Germany at €55,003 (≈US$74,800 in 2014), the United Kingdom at €62,343 (≈US$84,800 in 2014), the Netherlands at €46,990 (≈US$63,800 in 2014), and Estonia at €23,355 (≈US$31,700 in 2014), based on historical exchange rates at the time of each study.^14^ These disparities underscore the highly variable economic burden of MDR-TB treatment worldwide and highlight the need for tailored strategies and international collaboration to effectively mitigate this impact. By addressing these challenges with context-specific approaches and leveraging best practices from higher-income countries, Iran can enhance its management of MDR-TB, leading to better health outcomes and reduced economic strain on its healthcare system.

 XDR-TB, although rare, with only two patients identified in this study, imposed the highest per-patient cost among all TB categories. The average cost per XDR-TB case was I$21,946.5, resulting in a total expenditure of I$43,892.9. This elevated cost arises from the need for prolonged hospitalization, reliance on specialized second-line drugs, frequent follow-up, and management of severe complications. While the overall number of XDR-TB cases is limited, their disproportionately high financial burden underscores the critical importance of early detection, containment, and investment in preventive strategies. The global financial burden of XDR-TB treatment is profoundly high, showing significant variation across countries. Comparative data illustrate the range of costs: in South Africa, the cost per patient is US$26,392.0^21^; in the United States, the cost is significantly higher at approximately US$526,000.0 per patient^26^; the Russian Federation reports an average cost of US$37,800.0 per patient^27^; while in India, it is estimated at US$3,000.0 per patient.^17^ European countries also report high treatment costs, with Germany at €188,466, the Netherlands at €148,136, and Estonia at €23,355.^14^ In our study, the cost of treating XDR-TB was 30 times higher than that of DS-TB. Previous modeling studies from the USA, Asia, and South Africa have suggested that these costs can range from 40 to 200 times higher.^21^ This discrepancy might be attributed to several factors, including differences in healthcare infrastructure, availability of medical resources, socioeconomic conditions, and variations in study design and methodology. A clear understanding of these factors is crucial for developing more effective and equitable global strategies to manage XDR-TB.

 The economic burden of TB treatment in Iran presents unique challenges and places a significant financial strain on the healthcare system. Although the cost per patient for DS-TB is relatively lower, due to the high number of cases, it still represents a considerable portion of the total expenditure. In contrast, MDR-TB and XDR-TB incur substantially higher costs per patient, reflecting the intensive resources required for their treatment. These findings highlight the necessity for targeted strategies, enhanced resource allocation, and strengthened international collaboration to effectively reduce the financial burden of TB in Iran.

 The majority of the economic burden of DS-TB in Iran is attributed to direct medical costs, amounting to I$441.9 (66%). This dominance of medical costs highlights the need for efficient healthcare strategies that can reduce unnecessary expenses. Comparable trends in other countries also report significant economic burdens due to direct medical costs. For example, in Vietnam,^22^ direct medical costs are US$2,925.0 (68%); in Zimbabwe,^23^ they are US$1,765.0 (49%); and in China,^15^ they amount to US$ 6,316.0 (76%). In contrast, some countries report higher indirect costs. In Indonesia,^13^ indirect costs are US$1,344.0 (48%); in the Philippines,^25^ they are US$1,384.6 (59%); and in Laos,^28^ they reach US$ 1,324.0 (65%).

 These findings underscore the financial strain posed by TB treatment and the necessity for context-specific strategies. In Iran, optimizing direct medical costs could greatly alleviate the burden on the healthcare system. Comparative data from other countries demonstrate similar trends, highlighting the universal challenge of managing TB-related expenses. However, in some countries, the significant indirect costs, often linked to workplace absenteeism and non-medical expenses, targeted strategies are required to address productivity losses and enhance insurance coverage. Overall, these findings highlight the urgent need for tailored interventions and international collaboration to effectively manage and reduce the economic impact of TB.

 The average pre-diagnosis cost per individual suspected of having TB in Iran is I$22.4. For example, pre-diagnosis costs were US$97.6 in Ethiopia,^29^ US$63.8 in Pakistan,^19^ and US$402.0 in India.^17^ In these countries, more than 60% of pre-diagnosis costs were attributed to indirect costs, primarily workplace absenteeism. This highlights the necessity of addressing both direct and indirect costs in TB management to create a more sustainable healthcare system. By focusing on reducing direct medical costs and productivity losses, Iran can improve patient outcomes while reducing the financial strain on its healthcare infrastructure.

 The demographic and socioeconomic distribution of TB cases in Iran also reveals significant disparities that shape disease burden. Notably, males account for the majority of TB cases in Iran (57%), consistent with global trends showing higher TB prevalence among men. According to the Global Tuberculosis Report 2024,^1^ 55% of TB cases worldwide are reported in males, reinforcing this pattern. Similar trends are observed in India, where men constitute around 58% of TB cases,^17^ indicating higher vulnerability among males due to socioeconomic and lifestyle factors. Likewise, in South Africa, men represent approximately 56% of TB cases,^21^ further supporting this global pattern. This gender disparity may be attributed to factors such as differential exposure to high-risk environments, occupational hazards, and potential inequalities in access to healthcare services. Recognizing these patterns is essential for devising targeted interventions and public health strategies to effectively combat TB among vulnerable populations.

 In Iran, 70.0% of TB cases are reported from urban areas, with the remaining 30.0% from rural regions. This urban predominance aligns with global observations. For instance, approximately 67.0% of TB cases in Brazil are concentrated in urban areas,^30^ while in India, around 70.0% of TB cases occur in urban settings.^17^ In Zambia, a study highlighted a higher prevalence of recurrent TB cases in urban areas (15.3%) compared to rural areas (11.3%).^24^ Urban areas often present a paradox: despite better healthcare infrastructure, crowded living conditions and increased social interactions contribute to higher disease transmission rates. This disparity between urban and rural TB cases highlights the need for targeted public health strategies that address the distinct challenges of TB control in both settings. Comprehensive approaches that integrate urban healthcare advancements with outreach programs in the outskirts of cities are essential to effectively combat TB on both national and global levels.

 Regarding marital status, married individuals present the majority of TB cases in Iran (62.0%), followed by single (21.0%) and widowed individuals (13.0%). This distribution reflects the age demographics and societal structure in Iran, suggesting that the higher prevalence of TB among married individuals may be partly due to age, as older age populations are more likely to be married and at higher risk for developing TB. Similar trends are observed in Bangladesh, where married individuals also form the largest proportion of TB cases, indicating similar socio-cultural influences on TB epidemiology.^31^ Studies from other regions further suggest that marital status can influence TB prevalence; for example, in Nigeria, married individuals were found to have higher TB rates due to socio-economic factors and living conditions that may increase exposure risk.^32^ Likewise, research conducted in Pakistan indicated a higher incidence of TB among married individuals, which was attributed to household crowding and limited access to healthcare.^19^ Understanding these socio-cultural dynamics is essential for developing targeted interventions to effectively reduce TB transmission in these populations.

 In Iran, the age distribution of TB cases indicates that individuals over 65 constitute the largest affected group (29.0%), followed by the 25–34 age group (17.0%) and the 35–44 age group (15.0%). This pattern is consistent with global data from countries such as China, where elderly populations experience a higher prevalence of TB due to comorbidities and weakened immune systems.^15^ Similarly, a study in South Africa revealed significantly higher TB incidence among individuals over 60 years old, accounting for a substantial portion of cases.^21^ In India, a similar age distribution was observed, with the highest prevalence of TB among those aged 65 and above.^17^ The increased vulnerability of older adults to TB can be attributed to age-related immune decline, the greater likelihood of comorbidities, and potential delays in diagnosis and treatment. Understanding these age-related patterns is crucial for developing tailored interventions and public health strategies to effectively reduce the burden of TB in elderly populations.

 Educational data further reveal that 42.0% of TB patients in Iran are illiterate, and 23.0% have only an elementary education. This finding aligns with global findings indicating that low literacy is closely linked to higher TB prevalence, primarily due to limited access to health information and healthcare services. For example, a study in Ethiopia identified illiteracy as a significant predictor of TB risk, as individuals with no formal education had a higher risk of developing the disease.^29^ Similarly, research in Bangladesh indicated that low literacy was associated with delayed diagnosis and treatment of TB, thereby contributing to ongoing transmission rates.^31^ Addressing educational inequalities through targeted public health campaigns and improving access to healthcare services in low-literacy populations is therefore essential for strengthening TB control.

 In terms of disease type, pulmonary TB cases account for 81.0% of cases in Iran, consistent with global patterns in which pulmonary TB predominates due to its higher transmissibility and clinical severity. Similar patterns are reported elsewhere. In Indonesia, pulmonary TB constitutes around 78.0% of TB cases, emphasizing the need for robust detection and treatment protocols.^13^ In India, pulmonary TB represents approximately 75.0% of all TB cases, underscoring the global challenge of controlling this highly infectious form of TB.^17^ Moreover, research from Nigeria indicates that pulmonary TB accounts for about 80.0% of cases, reflecting similar patterns across different regions.^32^ Addressing the prevalence of pulmonary TB requires comprehensive public health strategies, particularly early detection, timely treatment, and consistent follow-up mechanisms, to prevent transmission and ensure successful treatment outcomes.

 DS-TB accounts for 98.2% of cases in Iran, while MDR-TB and XDR-TB constitute 1.7% and 0.2%, respectively. This distribution reflects the broader global challenge of managing drug-resistant TB, which requires extensive resources and specialized treatment protocols. For example, MDR-TB prevalence is considerably higher in Russia (about 10.0%), highlighting variations based on regional healthcare capabilities.^33^ Similarly, in South Africa, MDR-TB accounts for approximately 14.0% of TB cases, with XDR-TB making up about 2.0%,^21^ while Indonesia reports a higher prevalence of MDR-TB at approximately 7.0%.^13^ These higher rates in countries with a high TB burden highlight the pressing need for robust diagnostic capabilities, individualized treatment plans, and sustained public health interventions to mitigate the impact of drug-resistant TB.

 Effective management of drug-resistant TB necessitates comprehensive public health strategies, including timely diagnosis, individualized treatment regimens, and continuous monitoring to prevent the spread of resistant strains. Addressing these challenges is crucial for global TB control efforts.

 Our analysis demonstrates the multifaceted nature of TB in Iran, which broadly aligns with global trends while also showcasing specific regional challenges. Addressing these disparities through targeted healthcare strategies, supported by international collaboration, can significantly reduce the TB burden in Iran.

 The economic burden of TB in Iran, particularly across DS-TB, MDR-TB, and XDR-TB, underscores the urgent need for targeted, context-specific approaches to cost management. The average cost per patient for DS-TB, MDR-TB, and XDR-TB places a substantial financial strain on Iran’s healthcare system, with significant disparities observed across various regions globally. High direct medical costs, particularly for DS-TB, highlight the importance of improving healthcare resource allocation and optimizing treatment protocols. In contrast, the indirect costs predominated in certain countries underscore the need for strategies that mitigate productivity losses and non-medical expenses. In Iran, socio-economic factors such as gender, age, education, and urban–rural distribution also play critical roles in shaping TB prevalence and treatment outcomes. Greater international collaboration and the adoption of best practices from other countries, especially those with more advanced healthcare systems, can facilitate the development of comprehensive solutions to address the multifaceted challenges posed by TB. By leveraging these global insights and enhancing resource efficiency, Iran can substantially alleviate the economic burden of TB and improve overall public health outcomes.

 One important limitation of this study is its reliance on a prevalence-based cost-of-illness approach. While this method offers a cross-sectional estimate of the economic burden of TB in 2024, it does not account for long-term costs such as premature mortality, chronic disability, or post-treatment complications, especially in MDR- and XDR-TB cases. In contrast, an incidence-based approach would provide a more comprehensive assessment by following patients over time and estimating lifetime costs and consequences. Such models are particularly valuable for capturing the economic impact of extended treatment durations, long-term disability, and productivity losses due to TB-related deaths. However, due to data constraints and the study’s objective, we adopted a prevalence-based framework to quantify the annual burden at the national level. Future research should aim to employ incidence-based or dynamic modeling techniques, such as Markov or microsimulation models, to better inform cost-effectiveness analyses, long-term policy planning, and financial protection strategies.

 Our findings remained robust across a range of plausible parameter values, as demonstrated in sensitivity analyses. The inclusion of uncertainty assessment enhances the generalizability and policy relevance of our results, especially for cross-country comparisons and resource allocation decisions under uncertainty.

 The sensitivity analysis revealed that productivity losses due to temporary disability and hospitalization costs were the most influential drivers of the total economic burden of TB in Iran. This finding aligns with global evidence^3^, which displays that indirect costs often exceed direct costs in TB care, especially among working-age populations in low- and middle-income countries. Variations in daily wages and absenteeism duration further affected the results, indicating the need for reliable labor market and epidemiological data in national cost models. Although direct costs such as DOTS implementation, RT-PCR, and GX testing contributed to the overall burden, their impact was relatively lower. PSA using Monte Carlo simulations confirmed the robustness of the total cost estimate (mean: I$26.4 million; 95% CI: I$21.7–31.2 million). However, a key limitation is the lack of sensitivity analysis on the discount rate applied to estimate productivity losses due to premature mortality. Although a 5.0% rate was applied in line with standard recommendations,^8^ exploring a range of discount rates (e.g., 3.0%-7.0%) would have provided a more comprehensive view of long-term cost variability. Overall, the analysis suggests that targeting major cost drivers, particularly those related to lost productivity and hospitalization, can enhance the cost-efficiency of national TB control efforts in Iran.

 It is important to acknowledge several limitations that may affect the interpretation of our findings. First, the study relies on cost estimates that, despite adjustment for inflation and expert validation, are primarily derived from previously published data, which may not fully capture current regional variations and actual expenditures in 2024. Direct collection of recent field data or patient surveys would provide more precise, context-specific estimates, and we recommend that future research incorporate such primary data to enhance the accuracy and relevance of economic burden assessments. Second, the study does not account for potential indirect costs related to intangible factors, such as the psychological impact on patients and caregivers or long-term socioeconomic consequences. Third, the analysis is constrained by the availability of comparable data from other countries, which may introduce differences in cost structures and healthcare practices. Finally, the study focuses on a single year, limiting the ability to assess long-term trends and changes in the TB-related economic burden. Future research should also address these limitations by incorporating more comprehensive and longitudinal data to provide a deeper understanding of the economic impact of TB.

**Highlights**

- This study provides the first comprehensive estimate of the economic burden of tuberculosis (TB) in Iran using a prevalence-based approach from a societal perspective.
- In 2024, the total TB-related cost was approximately I$10.04 million, with 47% incurred during the pre-diagnosis phase and 53% during the post-diagnosis phase.
- Although infrequent, drug-resistant TB cases (MDR/XDR) generated significantly higher per-patient costs compared to drug-sensitive TB.
- Indirect costs, primarily due to productivity losses and premature mortality, constituted the largest share of the total burden (over 40%).
- The findings highlight the urgent need for investments in early detection, cost-effective diagnostics, and social protection policies to mitigate TB’s economic impact.

## Conclusion

 This study highlights the significant economic burden of TB in Iran, estimated at US$26.5 million in 2024, with direct medical costs forming the largest share. Treatment expenses varied across TB resistance patterns (i.e., DS-TB, MDR-TB, and XDR-TB). The findings underscore the need for efficient healthcare strategies to reduce costs, mitigate productivity losses due to temporary disability, and implement tailored public health policies. Additionally, demographic disparities, such as higher TB prevalence among males, urban residents, and older adults, highlight the importance of comprehensive public health approaches.

## Acknowledgments

 We would like to express our sincere gratitude to all those who contributed to this study. Special thanks go to the Management TB Department of the Ministry of Health for their invaluable support and cooperation throughout the research process. We also appreciate the staff and faculty of Tehran University of Medical Sciences for their dedication and assistance. Finally, we acknowledge all participating healthcare providers, whose contributions were essential to the success of this research.

## Competing Interests

 The authors declare no conflict of interests.

## Ethical Approval

 This study was approved by the Ethics Committee of Tehran University of Medical Sciences (Approval Code: IR.TUMS.SPH.REC.1402.170). Aggregated data were used, ensuring the anonymity and confidentiality of individuals. Although consent to participate was not required for aggregated data, we adhered to ethical guidelines by maintaining the privacy and confidentiality of the information. We confirm that all research procedures adhered to the principles outlined in the Declaration of Helsinki.

## Funding

 This research was funded by Tehran University of Medical Sciences (Grant ID: 9921434001). We sincerely thank the university for its invaluable support, which made this study possible.
